# Supplementary material for: Transcriptomic Insights into Mechanisms of Early Seed Maturation in the Garden Pea (Pisum sativum L.)
Source: Cells. 2020 Mar 23;9(3):779. doi: 10.3390/cells9030779 (PMC7140803; doi:10.3390/cells9030779)
Supplement: Supplementary file 1 [file cells-09-00779-s001.zip › Supplementary materials/FileS3.zip!/FileS2/Hormones - Results/Summary Sheets - Modules/Overexpression Spots/Report.pdf]

Overexpression Spots

landscape

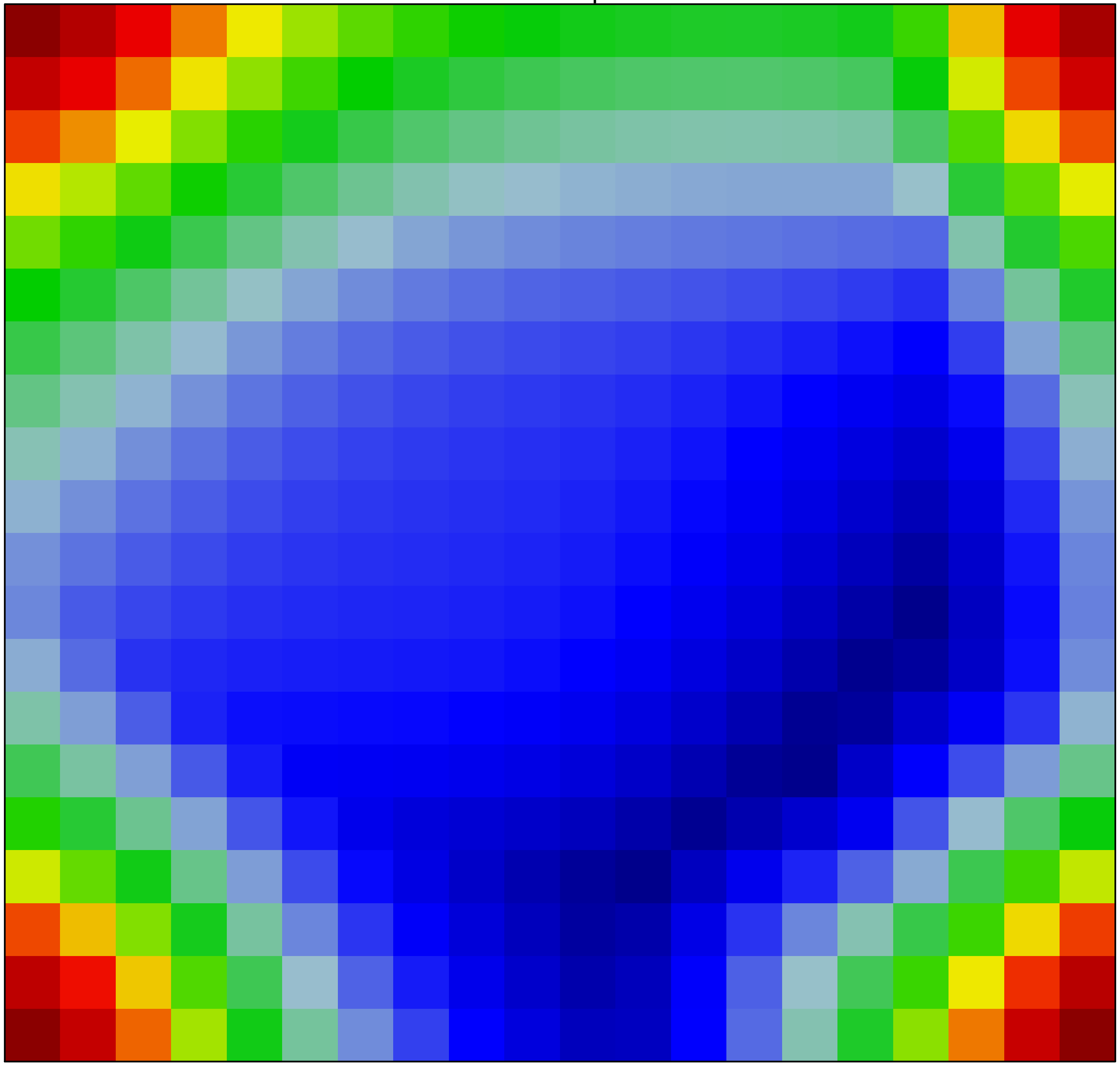

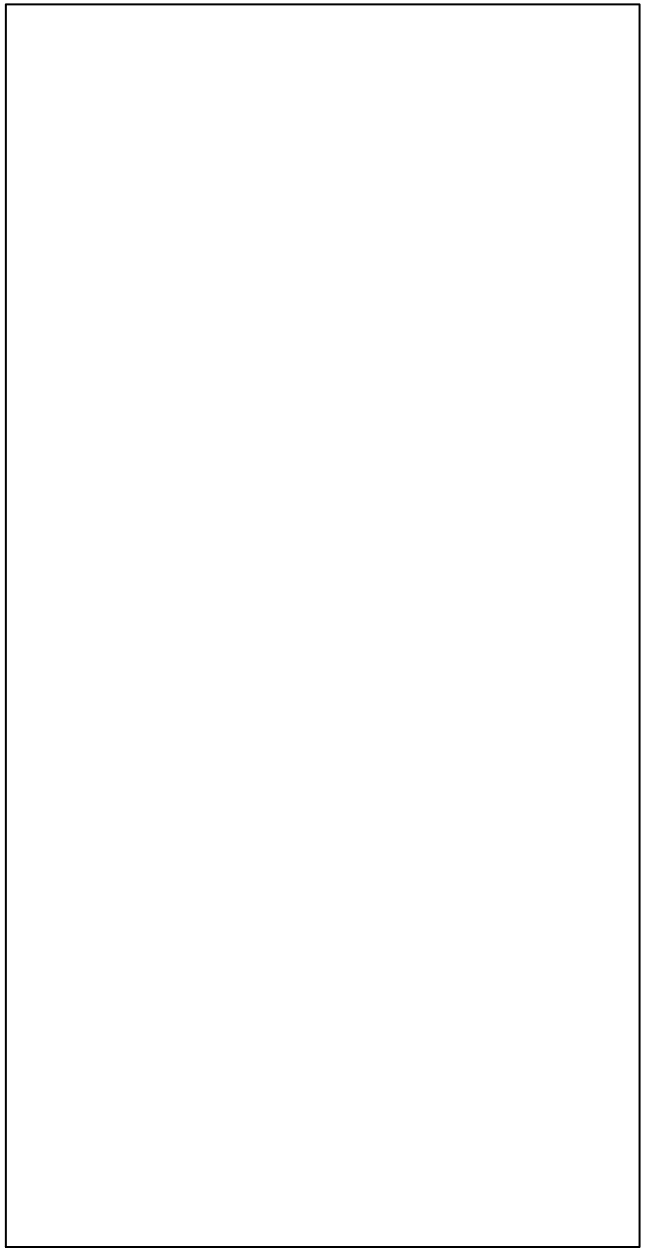

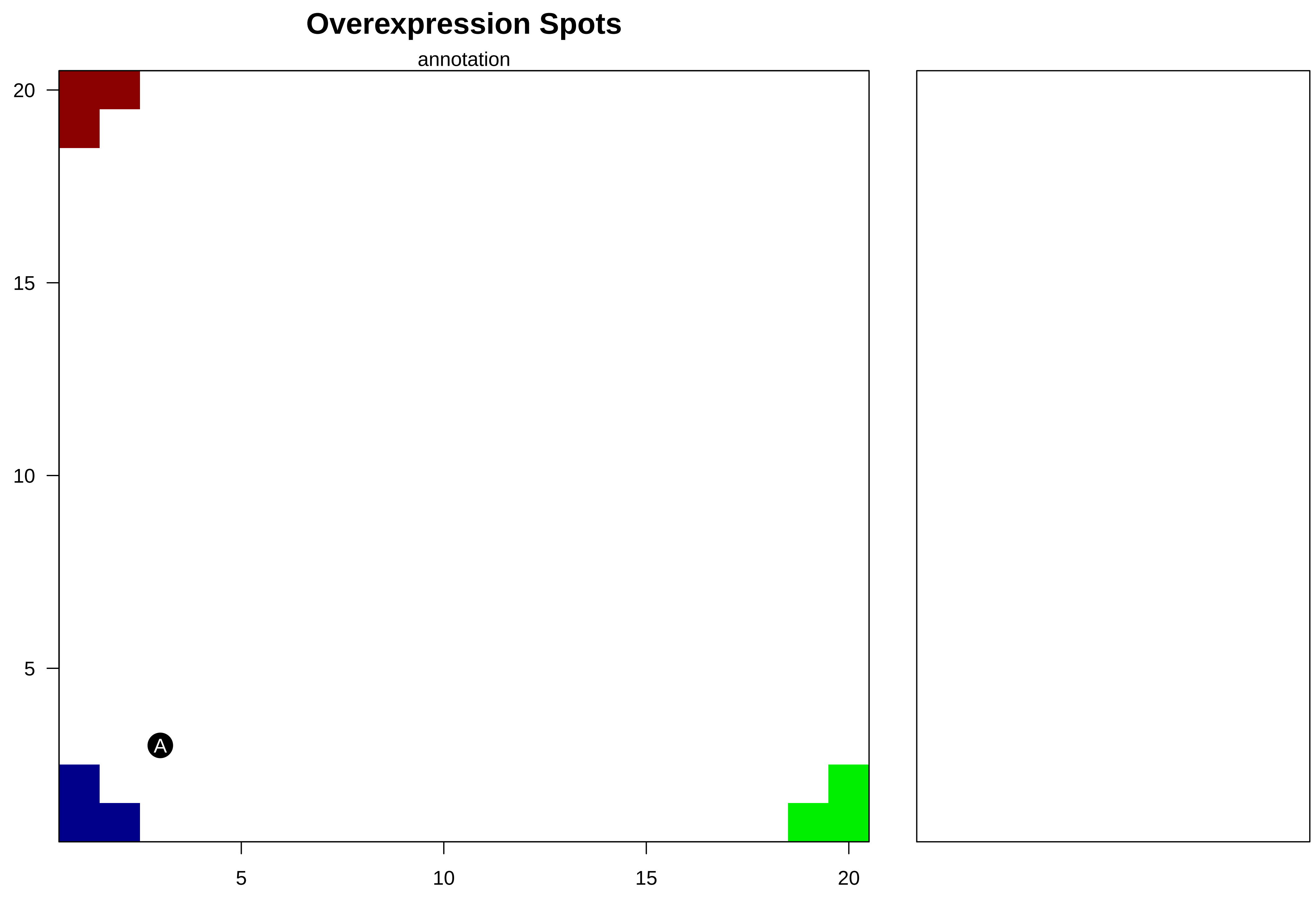
Overexpression Spots
annotation
20
15
10
5
A
5
10
15
20

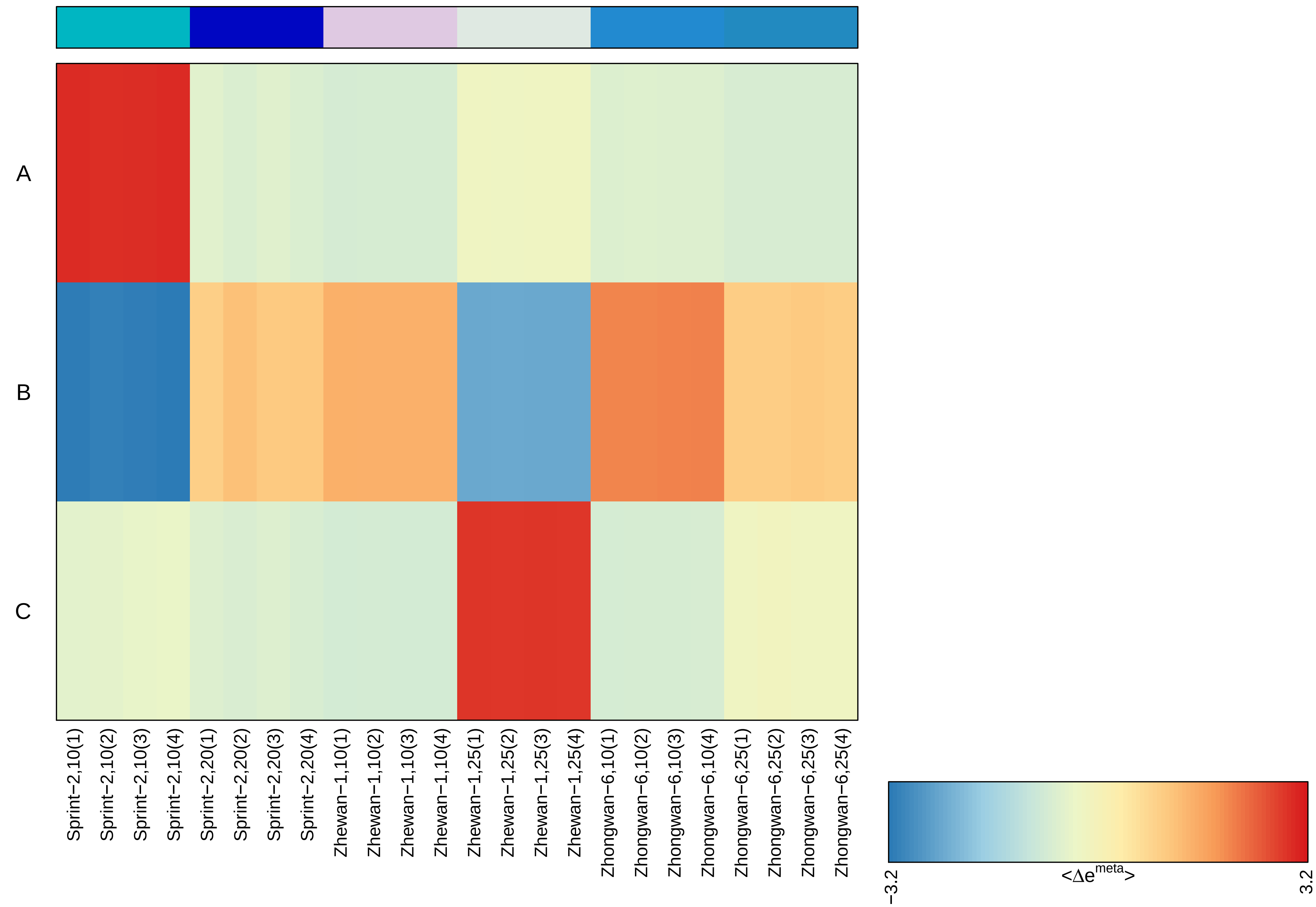
A
B
C
Sprint−2,10(1)
Sprint−2,10(2)
Sprint−2,10(3)
Sprint−2,10(4)
Sprint−2,20(1)
Sprint−2,20(2)
Sprint−2,20(3)
Sprint−2,20(4)
Zhewan−1,10(1)
Zhewan−1,10(2)
Zhewan−1,10(3)
Zhewan−1,10(4)
Zhewan−1,25(1)
Zhewan−1,25(2)
Zhewan−1,25(3)
Zhewan−1,25(4)
Zhongwan−6,10(1)
Zhongwan−6,10(2)
Zhongwan−6,10(3)
Zhongwan−6,10(4)
Zhongwan−6,25(1)
Zhongwan−6,25(2)
Zhongwan−6,25(3)
Zhongwan−6,25(4)
−3.2
<Δe^meta>
3.2

Overexpression Spots

Spot Summary: A

\# metagenes = 3
\# genes = 15

<r> metagenes = 1
<r> genes = 0.81
beta: r2= 2.13 / log p= −Inf

\# samples with spot = 4 (16.7 %)
Sprint−2(10) : 4 (100 %)

Overview Map

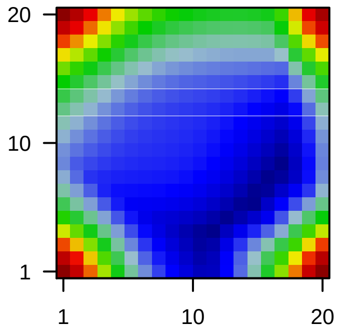

Spot

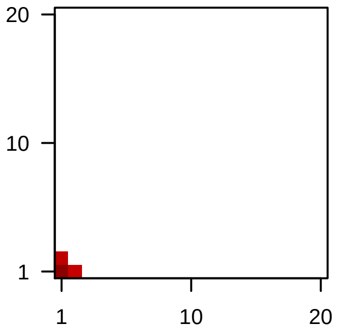

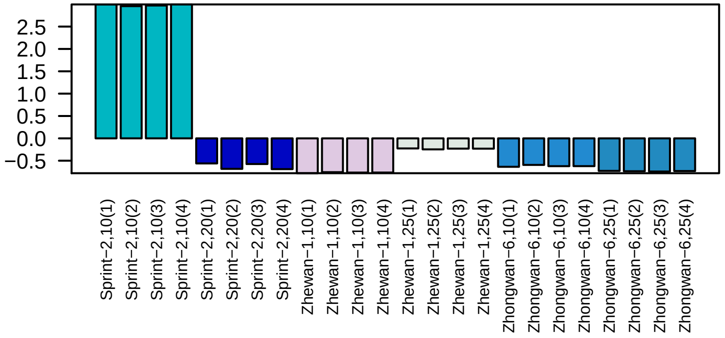

Spot Genelist

Rank	ID	max e	min e	r	Description	Symbol
1	TRINITY_DN1	15.48	−3.34	0.91		
2	TRINITY_DN3	15.03	−3.06	0.97		
3	TRINITY_DN4	4.64	−1.14	1		
4	TRINITY_DN8	3.6	−0.71	0.99		
5	TRINITY_DN4	3.56	−0.75	0.98		
6	TRINITY_DN8	3.06	−0.71	0.98		
7	TRINITY_DN6	2.31	−0.98	0.36		
8	TRINITY_DN3	1.81	−0.35	0.9		
9	TRINITY_DN3	1.66	−0.44	0.96		
10	TRINITY_DN1	1.51	−0.8	0.82		
11	TRINITY_DN5	1.42	−0.47	0.86		
12	TRINITY_DN1	1	−0.39	0.83		
13	TRINITY_DN2	0.93	−0.39	0.97		
14	TRINITY_DN1	0.85	−0.17	0.98		
15	TRINITY_DN2	0.69	−0.19	0.97		

Overexpression Spots

Spot Summary: B

metagenes = 3
genes = 8

<r> metagenes = 1
<r> genes = 0.44
beta: r2= 5.12 / log p= −Inf

samples with spot = 4 (16.7 %)
Zhongwan−6(10) : 4 (100 %)

Spot Genelist

Rank	ID	max e	min e	r	Description	Symbol
1	TRINITY_DN1	9.22	−1.86	0.59		
2	TRINITY_DN3	8.41	−2.58	0.39		
3	TRINITY_DN1	5.71	−13.31	0.98		
4	TRINITY_DN5	3.72	−1.76	0.48		
5	TRINITY_DN2	2.73	−3.16	0.82		
6	TRINITY_DN2	1.83	−1.59	0.52		
7	TRINITY_DN3	1.75	−1.32	0.82		
8	TRINITY_DN6	1.68	−1.13	0.58		

Overview Map

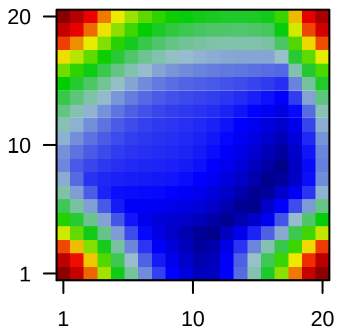

Spot

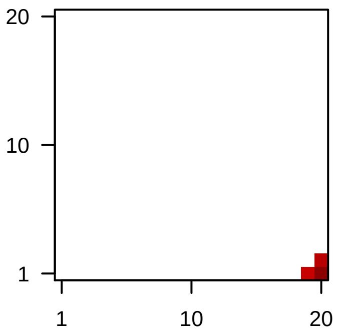

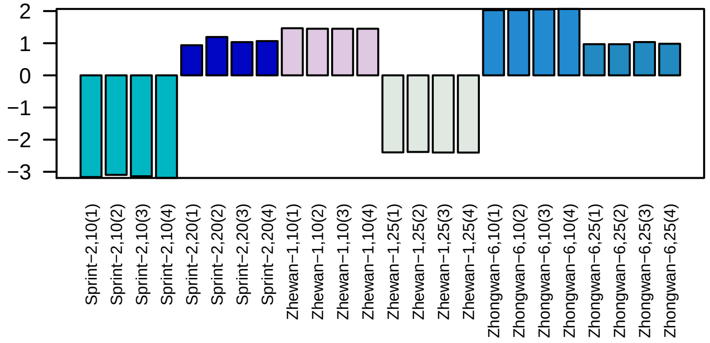

Overexpression Spots

Spot Summary: C

metagenes = 3
genes = 7

<r> metagenes = 1
<r> genes = 0.81
beta: r2= 1.02 / log p= −Inf

samples with spot = 4 (16.7 %)
Zhewan−1(25) : 4 (100 %)

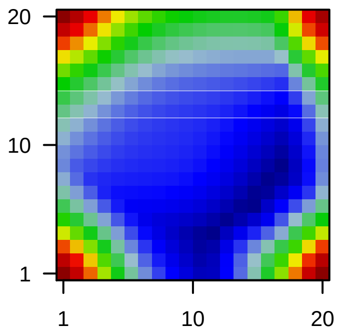

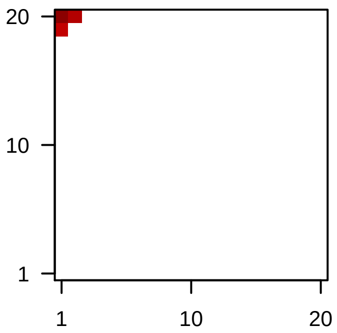

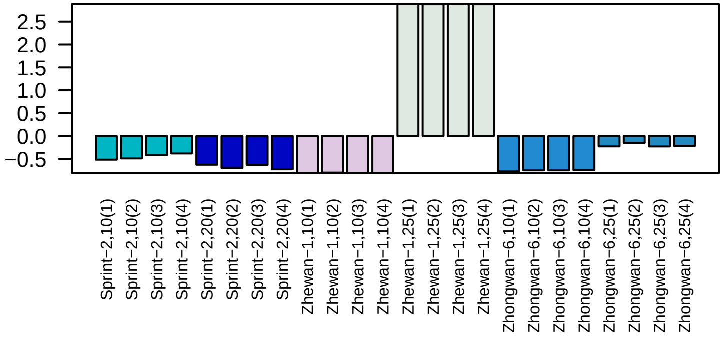

Spot Genelist

Rank	ID	max e	min e	r	Symbol	Description
1	TRINITY_DN3	15.54	−3.43	0.99		
2	TRINITY_DN1	1.68	−0.51	0.99		
3	TRINITY_DN1	0.79	−0.37	0.9		
4	TRINITY_DN5	0.75	−0.46	0.66		
5	TRINITY_DN5	0.74	−0.39	0.89		
6	TRINITY_DN4	0.59	−0.48	0.79		
7	TRINITY_DN1	0.52	−0.35	0.74		
